# Facile Synthesis of ZnO-CeO_2_ Heterojunction by Mixture Design and Its Application in Triclosan Degradation: Effect of Urea

**DOI:** 10.3390/nano12121969

**Published:** 2022-06-08

**Authors:** Antonia Cáceres-Hernández, Jose Gilberto Torres-Torres, Adib Silahua-Pavón, Srinivas Godavarthi, David García-Zaleta, Rafael Omar Saavedra-Díaz, Renan Tavares-Figueiredo, Adrián Cervantes-Uribe

**Affiliations:** 1 Laboratorio de Nanomateriales Catalíticos Aplicados al Desarrollo de Fuentes de Energía y Remediación Ambiental, Centro de Investigación de Ciencia y Tecnología Aplicada de Tabasco (CICTAT), DACB, Universidad Juárez Autónoma de Tabasco, Km.1 carretera Cunduacán-Jalpa de Méndez, C.P. Cunduacán 86690, TB, Mexico; 182a20212@alumno.ujat.mx (A.C.-H.); gilberto.torres@ujat.mx (J.G.T.-T.); adib.silahua@ujat.mx (A.S.-P.); rafael.saavedra@ujat.mx (R.O.S.-D.); 2 Investigadoras e Investigadores por México—División Académica de Ciencias Básicas, Universidad Juárez Autónoma de Tabasco, Villahermosa 86690, TB, Mexico; sgodavarthi@conacyt.mx; 3 División Académica Multidisciplinaria de Jalpa de Méndez, Carretera Cunduacán–Jalpa de Méndez, Universidad Juárez Autónoma de Tabasco, km 1, Col. La Esmeralda, Villahermosa 86690, TB, Mexico; david.garcia@ujat.mx; 4 CNPQ Conselho Nacional de Desenvolvimento Científico e Tecnológico, Brasília 86690, Brazil; renantf@infonet.com.br

**Keywords:** ZnO, CeO_2_, heterojunction

## Abstract

In this study, simplex centroid mixture design was employed to determine the effect of urea on ZnO-CeO. The heterojunction materials were synthesized using a solid-state combustion method, and the physicochemical properties were evaluated using X-ray diffraction, nitrogen adsorption/desorption, and UV–Vis spectroscopy. Photocatalytic activity was determined by a triclosan degradation reaction under UV irradiation. According to the results, the crystal size of zinc oxide decreases in the presence of urea, whereas a reverse effect was observed for cerium oxide. A similar trend was observed for ternary samples, i.e., the higher the proportion of urea, the larger the crystallite cerium size. In brief, urea facilitated the co-existence of crystallites of CeO and ZnO. On the other hand, UV spectra indicate that urea shifts the absorption edge to a longer wavelength. Studies of the photocatalytic activity of TCS degradation show that the increase in the proportion of urea favorably influenced the percentage of mineralization.

## 1. Introduction

Triclosan (TCS) is a sterilizing agent commonly used in consumer products such as soaps, toothpaste, etc. [1,2], typically with a concentration between 0.1–0.3% [3]. Kim et al. conducted studies to determine the antimicrobial efficiency of TCS, and concluded that there was no difference between soap with TCS and soap without this compound [4]. They also showed that there is no other skin benefit from the molecule. On the contrary, frequent exposure to TCS can generate alterations in bacteria, making them resistant to the chemical [5,6]. Chen et al. corroborated the resistance of bacteria to TCS [7]. The TCS biodegradation cycle is long [8], and persists in wastewater [9]. TCS can be removed from wastewater by activated sludge, with 72–94% effectiveness. The remaining TCS will contact the environment, contaminating lakes, rivers, and groundwater; hence, the potential impact on snails, algae, fish, mammals, and even humans is unavoidable [10,11]. According to Dayan et al., breast milk contains between 100 to 2100 μg/kg of TCS [12]. China identified the presence of TCS in its population in quantifiable concentrations [13]. Elsewhere, throughout Latin America, wastewater contains TCS [14,15]. Therefore, scientists remove TCS using physical and chemical techniques. For example, solid adsorbents remove TCS efficiently but at high costs [16,17,18]. Enzyme degradation is also efficient but depends on pH [19,20] and the type of ion; otherwise, the enzyme is blocked [21,22]. Advanced oxidation techniques such as ozonation [23], Fenton Fe^2+^ -UVC oxidation [24], electro-Fenton [24], and photocatalysis can mineralize TCS [25]. Photocatalysis is an environmentally friendly process and takes advantage of the energy emitted by the sun [26]. TiO_2_ and ZnO are among the semiconductors most used for the degradation of pollutants in photocatalysis. ZnO is more economical and has better activity when compared to TiO_2_ [27]. Due to its unique optical and electronic properties, ZnO has become a potential photocatalyst for industrial applications [28]. However, it has a bandgap of 3.2 eV and absorbs in the ultraviolet region. To improve its photocatalytic properties, different approaches have been employed, such as doping [29], metal loading [30], and heterojunction [31]. The case of ZnO creating a heterojunction with another semiconductor has been widely studied. The heterojunction is defined as the interface between two semiconductors with unequal band structures, and helps to increase the lifetime of charge carriers [32] by modifying the absorption region and enhancing activity [33,34,35]. On the other hand, incorporation of CeO_2_ improves mineralization [36] and the possibility of forming heterojunction [37] The Zn-CeO_2_ heterojunction is characterized by absorption in the visible region, and a lower bandgap than ZnO [38]. A wide range of ZnO/CeO_2_ materials has been developed, including CeO_2_-decorated ZnO nanorods [39], highly crystalline nanocomposites [40], and nanofibers [41]. All these materials show higher activity than ZnO and CeO_2_. Despite these good heterojunction qualities, activity can be further increased by adding a third compound, leading to the synthesis of ternary systems such as ZnO/CeO_2_/Cu_2_O [42] and CuO/CeO_2_/ZnO [43]. In ternary systems, the design of mixture experiments can be studied to determine the relationship between the ratios of the compounds and their responses [44,45]. In this study, we investigate the use of urea to increase the degree of heterojunction of the Zn-CeO_2_ system, using a simplex-centroid mixture. Urea is used because of its ability to form stable complexes with the metal ions in the solution, which aid in the formation homogeneous catalyst powders [46]. For this reason, we posit that urea will favor the formation of the heterojunction (ZnO-CeO_2_).

## 2. Materials and Methods

### 2.1. Materials

All reagents used were of analytical quality and were used without any purification treatment: ethanol (99.9%, Sigma, St. Louis, MI, USA), zinc nitrate (97%, Sigma), cerium nitrate Ce(NO_3_)_2_ 6H_2_O (99.9%, Sigma), and urea CO(CNH_2_)_2_. (99.0%, Sigma). All experiments were performed using ultrapure water (18.2 MΩ cm^−1^) from a PureLab model Option-Q water purifier (Satellite Blvd., GA, USA).

### 2.2. Nitrogen Adsorption

The determination of the specific area, diameter, and pore volume of the catalysts was conducted by the N_2_ physisorption technique. It was performed on equipment of surface area measurement, MICROMERITICS TRISTAR 3020 II, Communications, GA, USA, at −196 °C). A 0.1 g sample was weighed and degassed for 3 h at 300 °C to remove impurities. Data were analyzed using the BET method (Brunauer, Emmet, and Teller).

### 2.3. X-ray Diffraction

X-ray diffraction analysis was used to determine the composition of the phases and estimate the powders’ crystallite size. X-ray diffraction (XRD) was performed using a Bruker D2 PHASER diffractometer (Borken, North Rhine-Westphalia, Germany) with a Co Kα radiation source (λ = 1.5418 nm) during an analysis with a duration of of 660 s. The analysis was carried out in the range of 20° to 80°. The JADE 6 database helped to complete the identification of the phase. The average size of the crystals in the catalysts was estimated using the Scherrer equation:

(D=0.9/Cosθ)

### 2.4. Diffuse Reflectance UV-Vis Spectroscopy (DRS UV-Vis)

The UV–vis diffuse reflectance spectra were performed on a Varían Cary 300 spectrophotometer (Varian Inc., Palo Alto, CA, USA), in the range of 800 to 200 nm, equipped with an integrating sphere. A BaSO_4_ compound with 100% reflectivity was used as a reference. The bandgap energy (*E_g_*) of the samples was estimated from the UV absorption spectra, considering that: α(E) where $\propto \left(E\right)\propto {\left(E-E_{g}\right)}^{m/2}$ is the absorption coefficient for a photon of energy *E* and *m* = 4 for an indirect band transition [47].

### 2.5. Catalytic Test

Photocatalytic degradation tests were carried out in a photochemical reactor provided with UV light irradiation (λ = 365 nm) using a mercury lamp (13 W). At a natural pH, the photocatalyst (0.5 g/L) was dispersed in 200 mL of a TCS aqueous solution (10 ppm). As an oxygen source, an airflow (3.2 L/min) was provided to dissolve 8.4 mg/L of oxygen. Before the lamp was turned on, the suspension was continuously stirred at 700 rpm for 60 min in darkness, to establish an adsorption–desorption equilibrium between photocatalyst and pollutant. The system was maintained by circulating water at room temperature, and confined in a dark crate with UV light protection. Approximately 3 mL of the suspension was sampled and filtered (nylon, 0.45 m) to determine the ACP residual concentration, using a UV-Vis spectrophotometer (Varían, Cary 300). The results obtained calculated the degradation percentage, the total organic carbon percentage, the molar ratio of mineralization, and catalytic activity. Conversion percentages were determined using the following equation:

x(%)=([A°]−[A])/[A°]

where [A°] (ppm) is the concentration when the light is turned on to start the photodegradation process, and [A] (ppm) is the concentration after one hour under irradiation. Total organic carbon (*TOC*) was used. This is a global parameter to assess pollution elimination in water. Shimadzu *TOC*-VCHS analyzer equipment (Shimadzu Corp., Tokyo, Japan), the *TOC*-LCSN model, was employed to determine the *TOC*. The following equation determines the percentage of *TOC*:

TOC(%)=([TOC°]−[TOC])/[TOC°]

where [TOC°] (ppm) is the total organic carbon measured when the light is turned on to start the photodegradation process, and [TOC] (ppm) is the total organic carbon measured after one hour under irradiation. The efficiency corresponds to the total organic carbon conversion in $(mmol_{C}\;/L$) to UV degradation.

### 2.6. Simplex-Centroid Mixture

**Figure 1:**
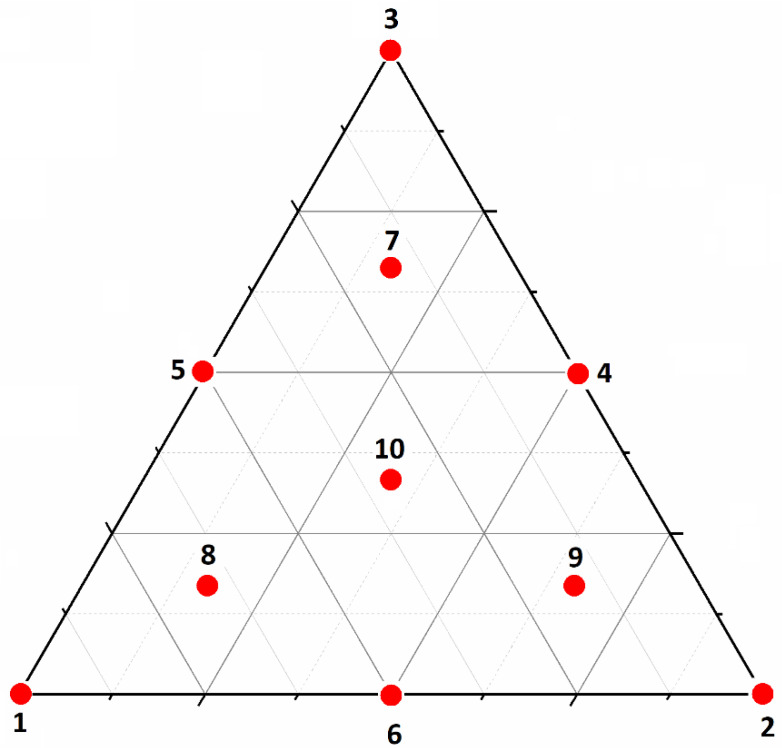
Three-factor simplex-centroid mixture design with three levels corresponding to urea, zinc nitrate, and cerium nitrate.

The simplex-centroid mixture is an experimental design used to understand the effects of the different components of a mixture with a minimum of experiments and an efficient mapping of the experimental space. The experimental space consists of different points distributed over an equilateral triangle, representing the proportions of the components of a mixture. The sum of the ratio of all the components of the mixture is equal to 1, or 100% [48]. This was the experimental design used to determine the effect of the proportions of ZnO, CeO_2_, and urea on the photodegradation of TCS. With the presence of constraints (i.e., $x_{i}=x+y+z=100\%$), the degree of freedom of the system was reduced from 3 to 2. Out of 10 samples studied, samples 1–3 represent the vertices in the ternary diagram, 4–6 represent the edges of the diagram with binary mixtures, while 7–10 are the ternary experimental space: see Figure 1. The experimental variation of the same sampled points was determined by synthesizing sample 10 three times: see Table 1.

The procedure consisted of dissolving the required amount in 15 mL of ethanol. The solutions were mixed and remained agitated for 1 h at 500 rpm. Subsequently, the solvent was removed in an oven at 80 °C for 12 h. The product obtained was solid, and received heat treatment at 500 °C for 3 h at 2 °C/min. Calculations were made to obtain 5 g of each sample. The amounts of each compound are shown in Table 1.

### 2.7. Temperature Programmed Oxidation (TPO)

The TPO study was carried out in a BELCAT-3000 apparatus (Bel-Japan, Tokyo, Japan) using a thermal conductivity detector (TCD), and 0.1 g of catalyst. In these experiments, the flow rate of the 5%/O_2_/95% He mixture was 10 mL/min, and the heating rate was 10 °C/min. Finally, the spectra were recorded from room temperature to 500 °C.

## 3. Results and Discussion

### 3.1. Nitrogen Adsorption/Desorption

**Figure 2:**
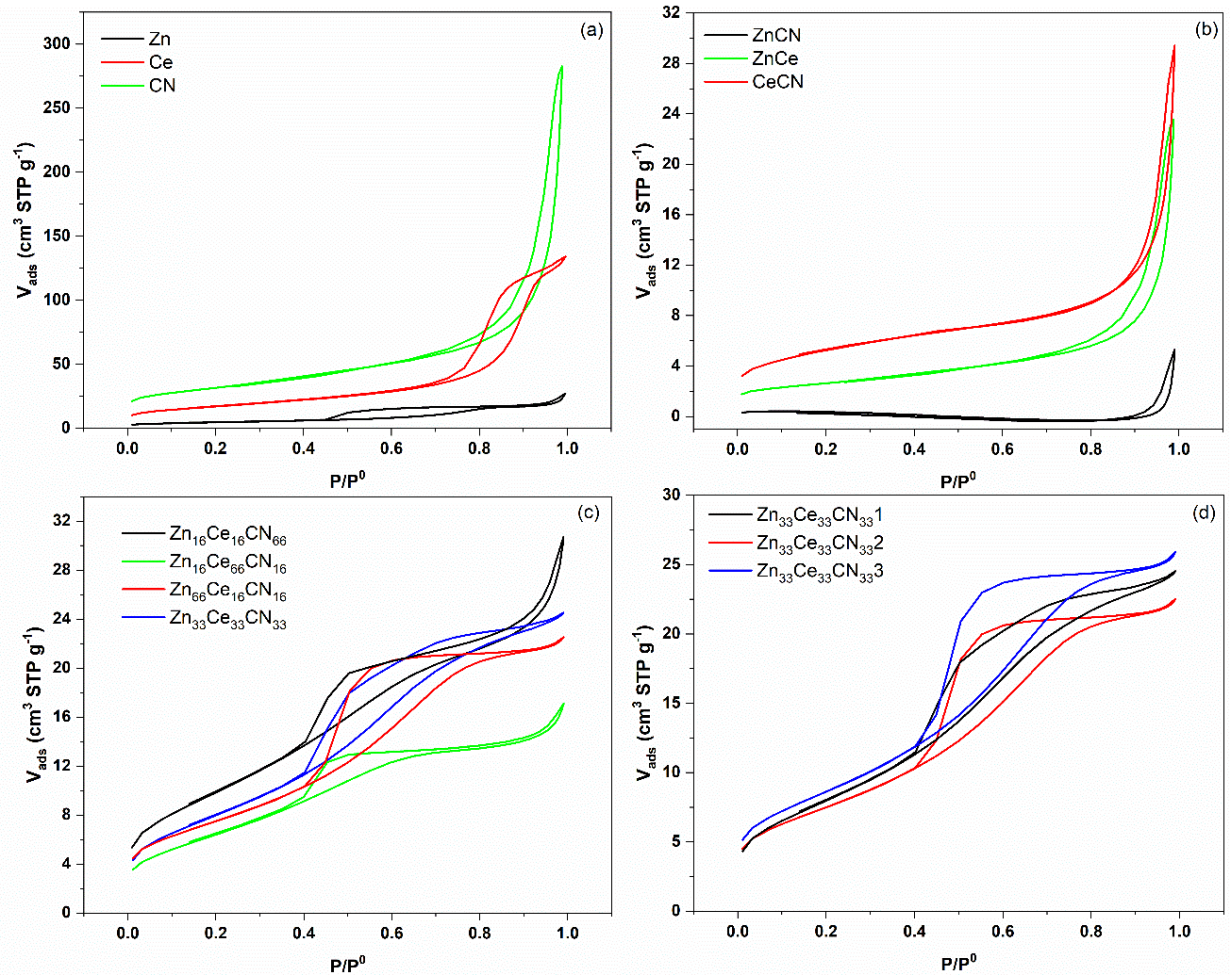
Nitrogen adsorption/desorption processes of pure (**a**), binary (**b**), ternary (**c**), and replica samples (**d**).

Figure 2 represents the results of the nitrogen adsorption/desorption process. All samples present type IV isotherms, characteristic of the adsorbent/adsorbate interaction between molecules in a condensed state. The hysteresis cycle is unique in each sample; it allowed us to identify the predominant pore types. The CN sample exhibits an H3−type hysteresis [49,50]. It presents two distinctive features: (i) the adsorption branch resembles a type II isotherm, and (ii) the lower boundary of the desorption branch is usually located at the cavitation-induced p/p0. Loops of this type are characteristic of non-rigid aggregates of plate-like particles. The sample Ce and Zn [51] have an H2(b)−type loop, and pores with large neck width. Binary (ZnCN, ZnCe, and CeCNN) and ternary material (Zn_16_Ce_16_CN_66_, Zn_16_Ce_66_CN_16_, and Zn_66_Ce_33_CN_33_) showed an H2(b)−type loop with the possible presence of surface blocked pores. Finally, the replicas (Zn_33_Ce_33_CN_33_) also present an H2(b)−type loop. Loop type and characteristics are taken from Tommes, M. et al. [52].

Surface areas were discussed to determine the influence of urea: see Table 2. The CN sample obtained the highest surface area, followed by Ce and Zn. As for the mixtures, zinc has the largest influence on surface area. It is probably the crystallinity of zinc that affects this property. On the other hand, there were similar areas across all of the replicate samples; therefore, there is a good reproducibility.

### 3.2. X-ray Diffraction

**Figure 3:**
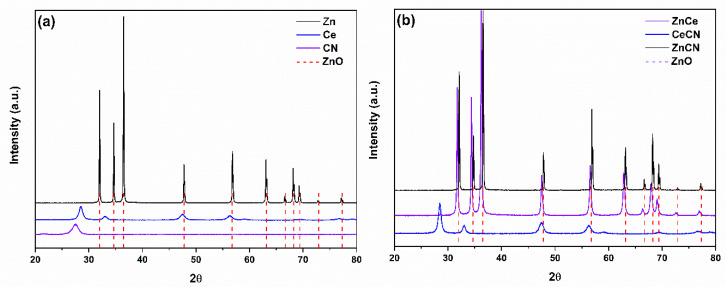
Diffraction patterns of (**a**) pure and (**b**) binary compounds.

Figure 3 shows the X-ray diffraction patterns of the pure and binary samples. Zn presented diffractions from the (100), (002), (101), (102), (110), (103), (200), (112), and (201) planes, corresponding to the hexagonal structure of zinc oxide. The diffractions of the zinc oxide were verified using JCPDS file No. 75-0576. The Ce sample has a cubic structure by diffractions in the planes (111), (200), (220), (311), (222), (400), (331), (420), and (422). The crystalline phase was corroborated using JCPDS file No. 65-2975. The CN sample showed diffractions at angles 24.5 and 13.1°, corresponding to the (002) and (100) planes of carbon nitride [53].

ZnCN has diffractions corresponding to the hexagonal structure of zinc oxide. The presence of a graphite phase is discarded; it is probably in an amorphous form. The diffraction intensity is lower and broader, compared to Zn. These two characteristics are typical of the formation of ZnO/C [54]. This product shows excellent activity in the visible region [55]. On the other hand, the CeCN sample exhibited the cubic structure of cerium oxide. The mixture of urea and cerium nitrate is explosive in the presence of an energy source [56]. Urea in contact with cerium nitrate decomposes to biuret and ammonia, then to isocyanic acid ((HNCO)_3_) at high temperatures [57]. On the other hand, urea promotes the nucleation of cerium oxide at low temperatures [58]. Therefore, the CeCN sample shows higher intensity diffractions concerning Ce. As for the ZnCe sample, it showed similar patterns to zinc oxide. However, it cannot be attributed to the hexagonal wurtzite structure of zinc oxide because it presents a left shift of 0.5°. According to the literature, the shift is caused by bond formation between -Ce-O- and -Zn-O [59], resulting in the ZnO-CeO_2_ heterojunction [60].

**Figure 4:**
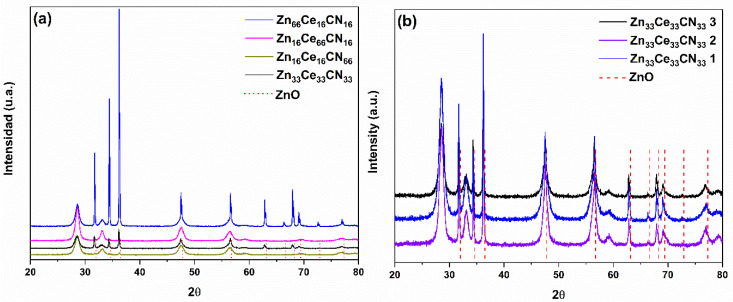
Diffraction patterns of (**a**) ternary mixtures and of the (**b**) replicate samples.

The results of the ternary samples are shown in Figure 4. In sample Zn_66_Ce_16_CN_16_, the hexagonal, cubic, and polymorphic structures of zinc oxide, cerium, and heterojunction (Zn-Ce) coexist. Sample Zn_16_Ce_66_CN_16_ is dominated by cerium oxide (cubic) and traces of Zn-Ce. Sample Zn_16_Ce_16_CN_66_ did not show the presence of carbon. However, the cubic structure of cerium oxide stands out. On the other hand, for Zn_33_Ce_33_CN_33_, the cubic structure of the cerium oxide and the heterojunction coexist. It is concluded that the displacement diffraction peak is a function of urea concentration, and may indicate a better interaction between ZnO and CeO.

The crystal sizes (D) are shown in Table 2. In the binary samples, the crystal size of zinc oxide is reduced; urea favors the crystal size of cerium. In ternary samples, higher urea concentrations increase the crystal size of cerium, and cause a reverse effect on zinc.

In Table 3, we analyze various literature sources related to heterojunction. Research articles 1, 6, 8, and 10 present X-ray diffractions with similar effects to the ternary samples. Additionally, paper 4 evidences displacement due to heterojunction. Moreover, the degree of heterojunction essentially depends on two factors: the precursors and the additives. If both precursors have the same organic or inorganic part, this favors the formation of a heterojunction.

### 3.3. Diffuse Reflectance UV-Vis Spectroscopy

**Figure 5:**
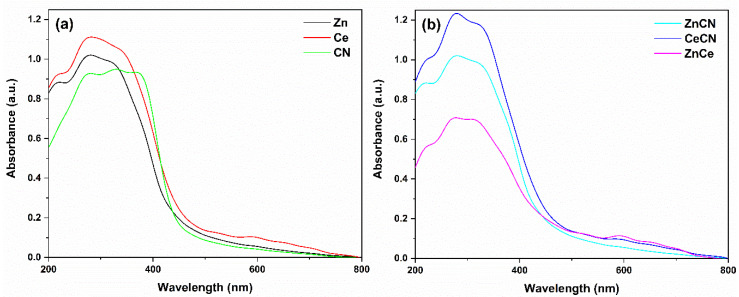
Absorption spectra of (**a**) pure and (**b**) binary samples.

The results of the UV absorption spectra are shown in Figure 5. In agreement with the literature, the Zn sample presents an absorption edge of 446 nm [71,72]. It also presents an absorbance at wavenumber 344 nm related to the photon absorption of Zn^2+^ [73]. Ce and CN show an absorbance edge at 428 and 460 nm, respectively [74,75]. The Ce sample presented two absorbances at wavenumbers of ≈ 290 and 260 nm, corresponding to the absorption of Ce^4+^ and Ce^3+^ oxidation states [76]. The CN sample presented an intense absorbance at 400 nm, about the degree of exfoliation of carbon nitride [77].

The ZnCN sample shows an absorption edge at 700 nm in the red region of the visible spectrum. According to the literature, carbon-doped zinc oxide shows an absorption edge in the red [78]. This might be induced by carbonaceous materials, which lead to better photocatalytic performance [55]. On the other hand, the ZnCe sample shows activation at 500 nm. According to Xiong et al., the interaction between cerium and zinc has a photosensitizing effect of absorbing the red [79]. Meanwhile, CeCN shows an absorption gap near 473 nm. This gap is slightly more significant than that of the Ce sample (461 nm). This slight enhancement is due to the interaction with nitrogen [80]. Thus, reduction reactions occur in ZnO, and oxidation reactions occur in CeO_2_.

**Figure 6:**
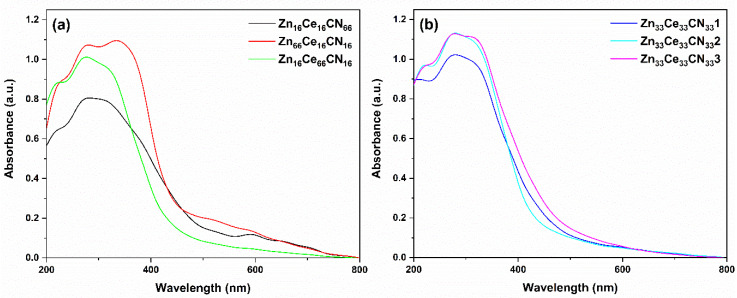
Absorption spectra of the (**a**) ternary and (**b**) replicate samples.

The results of the UV spectroscopy analysis of the ternary samples are shown in Figure 6. Zn_16_Ce_66_CN_16_ shows no absorption at wavelengths longer than 500 nm. The spectrum of Zn_66_Ce_16_CN_16_ showed absorptions at 459 nm and 700 nm. Zn_16_Ce_16_CN_66_ also showed two absorptions, at 542 nm and 700 nm. The sample Zn_33_Ce_33_CN_33_ showed activation at wavelength 446 nm, close to the values of the pure samples, while the replicates showed activation at 481 nm. Therefore, the reproducibility has a difference of 7.8%. The ternary samples possess high absorption in both the UV and visible regions. This indicates that the modified samples could benefit from both visible and UV responses.

Eg was estimated using the Tauc equation [81]. The Eg energies are given in Table 3. The ZnO sample presents an Eg in agreement with the literature [82]. Ce presents an Eg close to that reported in the literature [83,84]. Additionally, the Eg of CN agrees [85]. The heterojunction (ZnCe) obtained the lowest Eg value of the binary samples. Concerning the ternary samples, if the proportion of Zn is high, the Eg decreases. The Eg value is lowest in ternary mixtures with a higher proportion of urea (Zn_16_Ce_16_CN_66_). However, Eg increases with higher cerium ratios.

### 3.4. Catalytic Test

**Figure 7:**
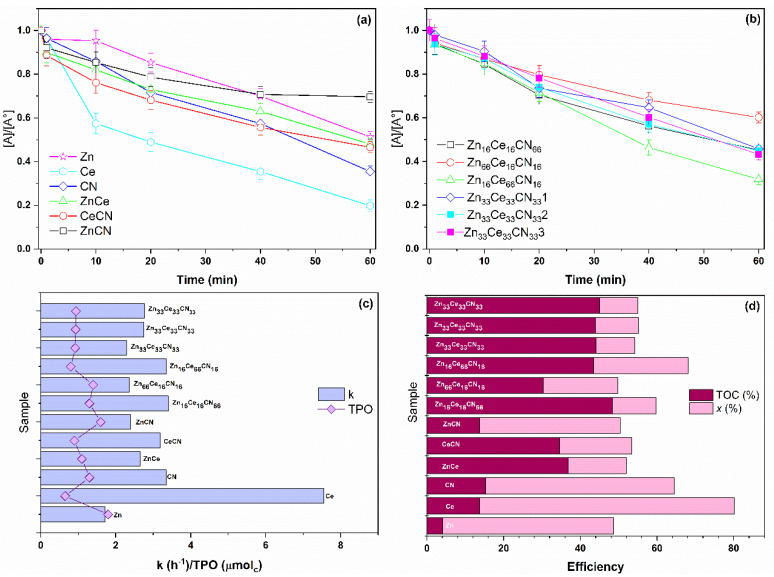
TCS degradation with (**a**) pure/binary, (**b**) ternary/replicates catalysts, (**c**) pseudo-first order constant/TPO, and (**d**) efficiency.

The photocatalytic activity of the samples was tested with a TCS conversion reaction under UV irradiation. The change in the normalized concentration as a function of time is shown in Figure 7a,b. All samples exhibited photocatalytic activity. The Zn, Ce, and CN samples showed conversions of more than 50%. However, they failed to mineralize the by-products: see Figure 7c. The binary samples obtained higher conversion values concerning CeCN, the removal of by-products being favored by cerium oxide. The ternary samples presented conversion values equal to or higher than Zn. In these samples, the degradation of by-products was efficient. The ternary samples are identified by the presence of the heterojunction of ZnO and CeO, and the increase in the amount of urea increased the mineralization. According to the reaction constant values, Ce obtained the highest value of the pure samples, CeCN of the binaries, and Zn_16_Ce_16_CN_66_ of the ternaries. The results of the TPO analysis show that Zn and CN present deposition of organic material on the surface, whereas Ce obtained the lowest deposition. The binary samples obtained lower deposited amounts compared to Zn. The deposition of organic matter in the ternary samples depends on two factors: urea and cerium.

The results were compared with articles related to the degradation of triclosan: see Table 4. From the table, it is understood that the pH and catalyst concentration influence the conversion and *TOC* percentage. The Zn and Ce samples of the current study presented an activity within the range reported in the literature. The binary combinations showed lower conversion but better *TOC* percent, even though the power source is of lower wattage in the current work. The ternary samples show similar conversions to those reported in Table 4, but higher *TOC* conversions than the binary and pure samples. The samples with heterojunction were shown to be highly efficient photocatalysts with high redox capacity [79].

**Figure 8:**
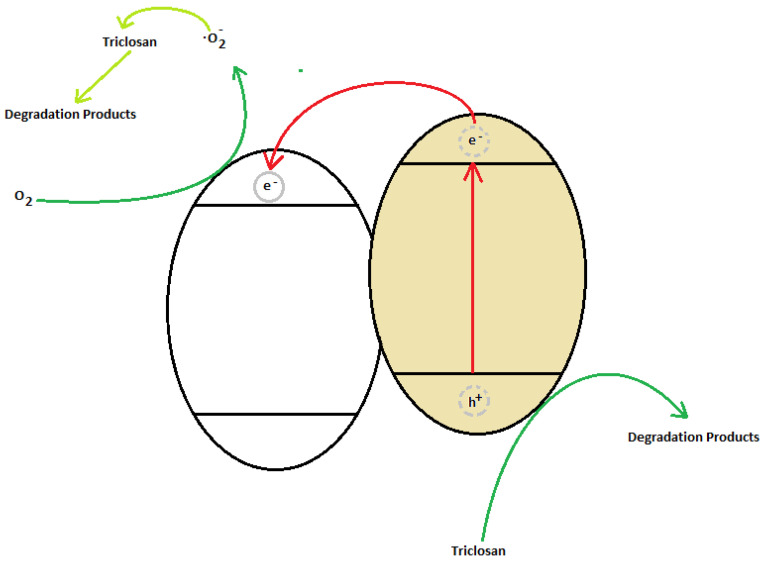
Z-scheme of electron transfer.

The photocatalytic mechanism considered as a semiconducting heterojunction was discussed according to the catalytic activity and characterization results. A possible Z-scheme electron transfer mechanism of the urea-assisted ZnO_2_−CeO_2_ composite catalyst ZnO_2_−CeO_2_ is proposed in Figure 8. The cerium oxide is excited under UV irradiation, producing electrons (e^−^) and holes (h^+^). The electrons rise to the valence band of cerium and are transferred to the conduction band of zinc. Oxygen interacts with this electron to reduce and form O_2_^−^. The oxidation of TCS takes place in the hole species (h^+^) and O_2_^−^.

Linear, quadratic, and cubic mathematical models [48,91] were fitted to the reaction efficiency (*TOC*) data using Statistica 12.0 software (Tulsa, OK, USA)[92]. The criteria for model choice were provided by the following statistical data: correlation coefficient, standard deviation, mean square F-test, and *p*-value. The *p*-value tests whether the model is significant or makes a significant additional contribution to explaining the response (*TOC*) when comparing mathematical models. The *p*-value criterion usually allows the model to be chosen if it is less than 0.05. Using this criterion, only the cubic model is suitable for modeling the response: see Table 5. If the *p*-value values of the three models were close, the correlation coefficient (R^2^) criterion would be chosen to select the model.

The coefficients of the selected model are shown in Table 6. It is inferred that the product obtained from precursor A achieves the most considerable response value (*TOC*) compared to the other two products (B and C): see Table 6. However, the combination of all three species has the most significant influence on the response. This suggests that efficiency is the synergistic result of the interaction between the species.

**Figure 9:**
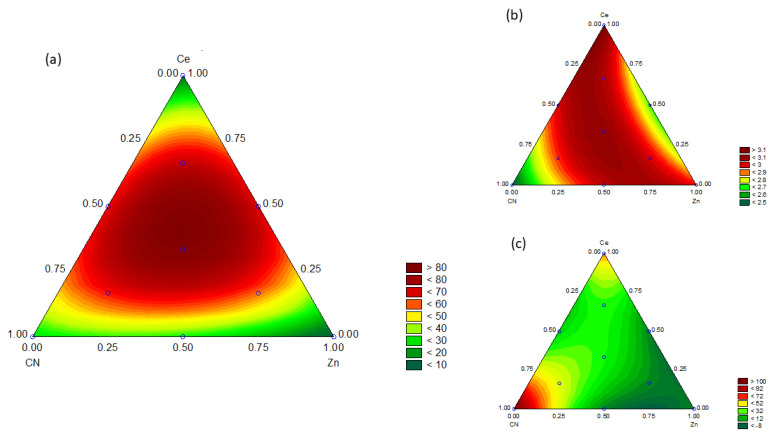
Contour plot projection of (**a**) mefficiency, (**b**) Eg, and (**c**) surface area.

The projected response surface in the contour plot of the experimental triangular space estimated by the mathematical model is shown in Figure 9. The contour plot illustrates the variations of the interactions in the response (*TOC*). The dark green regions represent low efficiencies, and the dark red regions represent high efficiencies. The optimal mixture covers a small area and is positioned just above the Zn_33_Ce_33_CN_33_ sample.

Figure 9 shows a contour plot projection of the response surface; b and c are contour plots of Eg and surface area responses.

The statistical analysis of Eg response and surface area is shown in Appendix A. The area with the lowest Eg (dark green color) covers only a fraction of the surface area. The region with the lowest Eg is found in the binary mixtures between Zn-Ce, heterojunction being the sample with the lowest Eg. The surface area values are homogeneous in all combinations. The optimum area value (dark red) is in and around the CN sample.

## 4. Conclusions

In this work, the ZnO−CeO_2_/urea system was synthesized by the solid-state combustion method to identify the role of urea in shaping heterojunction properties. According to the X-ray diffraction results, no additive, such as urea, is necessary for the formation of the heterojunction. The function of urea in the mixtures is more conducive to the formation of cerium oxide, while disfavoring heterojunction materials. The photocatalytic degradation of the TCS degradation reaction was tested. Zinc oxide and cerium oxide showed conversions higher than 50%. It is worth noting the activity of carbon nitride in the degradation; it presented a higher *TOC* conversion than zinc oxide and cerium oxide. The binary samples presented lower conversions with respect to their pure counterparts, but with greater degradation of by-products. In the ternary samples, the conversion of less than 50% persisted, although the degradation of by-products was greater than in the binary and pure samples. The mixture design helps us to understand the influence of the species. It also provides the most suitable composition for TCS mineralization.

## Figures and Tables

**Table 1:** Symbology of the samples and the proportion of each compound.

Sample	ZnO (%)	CeO_2_ (%)	Urea (%)	Symbology	Amount (g)
1	−	−	100	CN	9.8
2	100	−	−	Zn	11.4
3	−	100	−	Ce	12.5
4	50	50	−	Zn-Ce	6.3/5.7
5	−	50	50	Ce-CN	6.3/4.9
6	50	−	50	Zn-CN	5.7/4.9
7	16	16	66	Zn_16_Ce_16_CN_66_	1.9/2.1/6.5
8	66	16	16	Zn_66_Ce_16_CN_16_	7.6/2.1/1.6
9	16	66	16	Zn_16_Ce_66_CN_16_	1.9/8.4/1.6
10	33	33	33	Zn_33_Ce_33_CN_33_	3.8/4.1/3.2

**Table 2:** Surface area, crystal size, and Eg.

Sample	Área (m^2^/g)	D (nm) ^a^	D (nm) ^b^	D (nm) ^c^	Eg (eV)
CN	111	−	−	7.5	2.52
Zn	1	92.0	−	−	2.98
Ce	61	−	10.4	−	3.11
ZnCe	1	33.9	−	−	2.68
ZnCN	3	58.2	−	−	3.07
CeCN	19	−	13.0	−	2.98
Zn_16_Ce_16_CN_66_	36	30.1	9.4	−	2.82
Zn_16_Ce_66_CN_16_	24	30.5	11.3	−	3.14
Zn_66_Ce_16_CN_16_	4	82.0	10.6	−	2.93
Zn_33_Ce_33_CN_33_ 1	28	48.6	9.5	−	3.12
Zn_33_Ce_33_CN_33_ 2	29	55.5	9.5	−	3.14
Zn_33_Ce_33_CN_33_ 3	27	51.3	9.4	−	3.10

^a^ Data corresponding to ZnO. ^b^ Data corresponding to CeO_2_. ^c^ Data for C_3_N_4_.

**Table 3:** Precursors and additives for the synthesis of ZnO-CeO_2_ heterojunction.

#	Precursores		Aditivos	Método	Ref.
	Zn	Ce			
1	Zn(NO_3_)_2_	Ce(NO_3_)_2_	Lavander	Sunlight driven	[61]
2	Zn(aca)_2_	Ce(NO_3_)_2_	Polivinylpyrrolidine	Hydrothermal	[62]
3	Zn(NO_3_)_2_	Ce(NO_3_)_2_	NaOH	Hydrothermal	[63]
4	Zn(NO_3_)_2_	Ce(NO_3_)_2_	NaOH	Hydrothermal	[64]
5	Zn(NO_3_)_2_	Ce(NO_3_)_2_	NaOH	Hydrothermal	[65]
6	Zn(NO_3_)_2_	Ce(NO_3_)_3_	RhB	Hydrothermal	[66]
7	Zn(NO_3_)_2_	(N*H*_4_)_2_[Ce(NO_3_)_6_]	p-nitrophenol	Combustion	[67]
8	Zn(NO_3_)_2_	Ce(NO_3_)_2_	NaOH	Co-precipitation	[68]
9	Zn(AC)_2_	CeCl_2_	NaOH	Preciptation/impregnation	[69]
10	Zn(OAc)_2_	Ce(CH_3_CO_2_)	Etanolamine	Sol–Gel	[70]

**Table 4:** Comparison of articles with photodegradation of triclosan.

Catalyst	Concentration (g/L)	pH	TCS (ppm)	Conversion (%)	*TOC* (%)	Time (min)	Power (W)	Ref.
ZnO/Ca	0.3	10	10	99	−	200	125	[51]
ZnO	3.33	−	39	−	52	160	18	[86]
ZnO	1	7	5	25	−	360	4	[87]
CeO_2_	0.5	7	−	88	−	45	120	[88]
Nanorods g-C_3_N_4_	0.5	7	10	50	−	90	125	[89]
g−C_3_N_4_/MnFe_2_O_4_	0.2	9	9	93	44	60	−	[90]

**Table 5:** F-test for choosing the efficiency model (*TOC*).

Model	R^2^	SS	dF	MS	F	*p*-Value
Lineal	0.046	316.77	2	158.39	0.1684	0.8484
Quadratic	0.907	6261	5	1252	7.83	0.035
Cubic	0.941	6494	6	1082	7.96	0.058

SS: sum of squares. dF: degrees of freedom. MS: mean square. F: ratio F.

**Table 6:** Parameters of the Student’s test for the evaluation of the coefficients of the cubic model.

Factor	Coeff.	Std. Err	T-Statistic	*p*-Value
Urea (A)	28.61	11.26	2.54	0.084
Zinc Nitrate (B)	8.35	11.26	0.74	0.52
Cerium Nitrate (C)	13.89	11.26	1.23	0.31
AB	53.93	56.71	0.95	0.41
AC	181.02	56.71	3.19	0.049
BC	228.47	56.71	4.02	0.027
ABC	498.18	373.88	1.31	0.282

## Data Availability

Data are contained within article.

## Appendix A

The cubic mathematical model was fitted to the Eg response. The *p*-values and correlation coefficient support this: see Table A1.

**Table A1:** F-test to choose the Eg model.

Model	R^2^	SS	dF	MS	F	*p*-Value
Lineal	0.279	0.106	2	0.054	1.35	0.318
Quadratic	0.861	0.327	4	0.065	4.96	0.073
Cubic	0.915	0.348	6	0.058	5.41	0.097

SS: sum of squares. dF: degrees of freedom. MS: mean square. F: ratio F.

The coefficient values of the selected model are presented in Table A2. Of the pure compounds, C has a more significant influence on Eg. AB has a positive effect in the binaries. The BC combination has the opposite effect, increasing the value of Eg. Undoubtedly, the interaction of the three species (ABC) has a strong influence on Eg, as indicated by the value of the coefficient.

**Table A2:** Parameters of the Student’s test for evaluating the coefficients of the cubic model describing the Eg.

Factor	Coeff.	Est. Err	T-Statistic	*p*-Value
Urea (A)	2.488	0.101	24.86	0.0001
Zinc Nitrate (B)	2.963	0.101	29.614	0.0001
Cerium Nitrate (C)	3.147	0.101	31.449	0.0001
AB	1.182	0.503	2.347	0.101
AC	0.669	0.503	1.329	0.275
BC	−1.419	0.503	−2.818	0.066
ABC	4.595	3.321	1.383	0.261

The surface area is modeled with the cubic fit. This is indicated by the *p*-values and correlation coefficient, see Table A3.

**Table A3:** F-test for choosing the surface area model.

Model	R^2^	SS	dF	MS	F	*p*-Value
Lineal	0.568	6124	2	3062	4.592	0.054
Quadratic	0.912	9838	5	1967	8.26	0.031
Cubic	0.992	10699	6	1783	58.32	0.003

SS: sum of squares. dF: degrees of freedom. MS: mean square. F: ratio F.

The product obtained from urea calcination dramatically influences the response. This is indicated by the values of the coefficients of the sectioned model. It is worth mentioning that binary combinations negatively influence the area. Again, the interaction between species benefits this property: see Table A4.

**Table A4:** Student’s test to evaluate the coefficients of the cubic model describing the surface area.

Factor	Coeff.	Est. Err	T-Statistic	*p*-Value
Urea (A)	109	5.345	20.41	0.0002
Zinc Nitrate (B)	1.98	5.345	0.372	0.7351
Cerium Nitrate (C)	60.5	5.345	11.32	0.0014
AB	−213	26.91	−7.94	0.0041
AC	−272	26.91	−10.13	0.0020
BC	−118	26.91	−4.42	0.0214
ABC	941	177	5.30	0.0131
